# A multi-dimensional approach to the future of digital research infrastructure for systemic environmental science

**DOI:** 10.1016/j.patter.2024.101092

**Published:** 2024-11-08

**Authors:** Kelly Widdicks, Faiza Samreen, Gordon S. Blair, Susannah Rennie, John Watkins

**Affiliations:** 1 UK Centre for Ecology & Hydrology (UKCEH), Lancaster Environment Centre, Library Avenue, Bailrigg, Lancaster LA1 4AP, UK

## Abstract

Digital research infrastructure (DRI) for environmental science requires significant transformation to support the changing nature of science and utilize digital innovations. Numerous challenges prevent this change yet simultaneously pose exciting principles to drive the future of DRI. This opinion piece details a multi-dimensional approach toward these futures for the environmental community.

## Main text

Digital research infrastructure (DRI) is being designed, developed, and adopted for research and innovation^1^^,^^2^ and is fundamental to environmental science, including monitoring technologies for capturing environmental data, computational infrastructure to store and process this data, advances in models and methods to make sense of this (increasingly heterogeneous) data, and the provision of digital tools to enable collaborative science and communication with different stakeholder groups. Despite progress in this space, however, DRI needs significantly more time and investment to support the increasingly complex needs of environmental science as the world adapts to climate and ecological change.

Core to this is the transition in environmental science from studying environmental systems in silos toward understanding how systems interact through more *systemic environmental science*, i.e., science that gathers diverse data from these systems, analyses these together using models and methods across different areas of environmental science, and surfaces the interactions and feedbacks that are inherent in such systems. Simultaneously, there are significant and rapid innovations in digital technology that are not effectively utilized in environmental science to support these complexities. Cloud computing, for example, provides a wide range of on-demand, scalable, and elastic services that could offer essential building blocks for data management, parallel and distributed execution, data analyses, and visualization, cooperation, and sharing. Advancements have also been made in IoT (Internet of Things), data science, and AI (Artificial Intelligence) that could revolutionize the way we interact with our environment and make sense of the complex data of systems.

These two perspectives—the changing nature of environmental science and supporting innovations in digital technology—are driving the need for a step change in DRI to truly advance environmental science, but there are numerous challenges preventing this step change. For systemic science, we need to break down silos between environmental domains by supporting scientists’ collaboration and the integration of their insights, requiring data and other scientific digital assets (e.g., models, methods) that are FAIR (Findable, Accessible, Interoperable, Reusable).^3^ To ensure such collaboration is possible and enable decisions to be made on systemic science, DRI also needs to be usable by a range of stakeholders including non-experts, encouraging trust among these stakeholders by being open and transparent about the scientific process. Furthermore, to embrace digital innovations, we must ensure all digital assets are platform independent to adapt to new and emerging software architectures over time and to enable scalability to parallel or distributed architectures. As DRI uptake rises, we must also enhance the protection of assets (e.g., sensitive data) from cyber security breaches, ensure DRI is sustainable for long term use and maintenance, address DRI’s rising environmental costs, and enhance digital literacy in environmental science, ensuring that there are inclusive mechanisms for all scientists to benefit from digital innovations.

**Figure 1:**
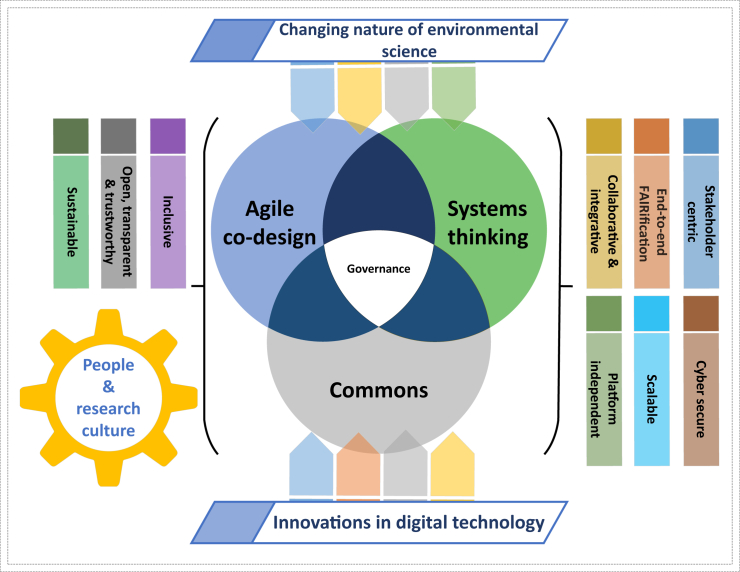
Digital research infrastructure futures: Our multi-dimensional research approach

While challenging, these potential barriers when flipped also form an exciting, ambitious, and transformative set of principles to achieve in DRI futures (Table 1). We recognize that we are a long way from realizing these DRI futures, yet given the climate and ecological crises, we must be ambitious to ensure DRI can meet the complex needs of environmental science. We therefore share our proposed research approach for the future of DRI for environmental science (Figure 1), governing and drawing on agile and co-design methods, systems thinking, and commons concepts while building teams for transdisciplinary DRI research. This approach has been scoped by our most recent research on UKCEH DRI projects, including within UK National Capability programs and the Floods and Droughts Research Infrastructure.^4^ We see this as a multi-dimensional approach (detailed below) that can support the environmental research community through the development of effective DRI, and by openly sharing our approach, we welcome and advocate for opportunities to collaboratively innovate with others in the community for this goal.

**Table 1:** A summary of key principles for DRI futures for environmental science

#	Principle	Description
1	collaborative and integrative	DRI should support collaboration between different stakeholders in environmental science. This should be underpinned by DRI innovations that can integrate underlying scientific assets and insights, offering a systemic view of our global environment and its changing nature.
2	end-to-end FAIRification	DRI should not only have FAIR data for open science, but also FAIR assets (e.g., models, methods, sensor infrastructure, workflows, tools) throughout the entire research life cycle from data collection to decision making. Technical and cultural barriers to the FAIRification of assets should be uncovered and overcome.
3	stakeholder centric	DRI should be designed to meet the needs of stakeholders in environmental science, including the range of scientists and decision-makers involved in the research. This includes advancing stakeholder-focused DRI designs, alongside offering DRI support and upskilling activities where necessary.
4	platform independent	DRI should be architecturally designed to be platform independent to allow research assets and activities to be ported from one technical infrastructure to another. This will ensure the DRI is flexible and extensible in adapting to new digital innovations over time.
5	scalable	DRI should scale to the compute needs necessary for the science, supporting complex data processing, methods and models on local machines, shared high performance computing or hybrid cloud facilities. This should support multiple and heterogeneous datasets in varying sizes and combinations for integrative science to be achievable.
6	cyber secure	It is important that DRI can offer sufficient levels of cyber security including protecting against cyber-attacks on the underlying infrastructure. These security and privacy concerns must be balanced against the desires to promote open, transparent, and accessible science.
7	inclusive	DRI should allow any stakeholder—regardless of their background, discipline, or expertise—to benefit equally from using DRI for their environmental science research or general enquiries. Diversity should be dutifully considered and celebrated in meeting stakeholder needs to support international environmental science and collaborations.
8	open, transparent, and trustworthy	Promoting openness and transparency in environmental science should be at the heart of DRI innovation, allowing all stakeholders to see the assets used and decisions made in the entire research life cycle. Any uncertainty in the science should be clear, growing trust amongst stakeholders in the science insights, outputs, and resultant actions.
9	sustainable	DRI should be financially sustainable and maintainable long-term and environmentally sustainable in its design. Environmental impacts from DRI’s creation, use, and disposal should be transparent and minimized by responsible innovation, with environmental gains from the science outweighing DRI’s environmental costs.

Note that we do not see these DRI principles as standalone; they overlap, can be combined in different ways, and will inevitably change or be added to overtime as the needs of environmental science evolve.

### Applying an agile co-design method

For DRI’s success, we advocate for a method that marries the benefits of co-design and agile software development. By co-design, we refer to developing DRI *with* stakeholders, whereby stakeholders’ perspectives are viewed as just as important to the those in the development team.^5^ An agile approach then complements this by offering an iterative and continuous approach to software development, prioritizing “to satisfy the customer through early and continuous delivery of valuable software.”^6^ When combined, an agile co-design approach intrinsically involves stakeholders at each stage of the DRI development continuously over time. This includes, for example, gathering requirements for the development of tools in the DRI but also activities that surround the DRI in research culture, e.g., training activities for DRI use. Through this, we will ensure the DRI truly and inclusively meets the needs of all stakeholders in environmental science (e.g., scientists, software developers, data managers, policymakers, citizens, etc.)—prioritising activities with stakeholders that are deemed most important and making adaptions as science and technological innovations evolve. We will strive to gain a diverse set of perspectives with these stakeholder groups, use inclusive research practices, and follow best practices in the research community regarding stakeholders and data repositories within the DRI, e.g., TRUST principles for supporting trustworthiness among stakeholders^7^ and CARE principles for data collaborations with indigenous communities,^8^ extending these to assets more generally.

### Adopting a systems-thinking lens

Given the interconnecting and changing nature of the environment, we must move toward a systems-thinking lens that aims to make relationships and interlinkages between domains of environmental science and other connected disciplines (e.g., social science) explicit within DRI design and development. This lens specifically supports a system to be considered “as a whole,” enabling those using it to understand the elements of the system, expose their connections, consider alternative futures, and creatively redesign systems through adapting relationships between elements.^9^ With this, we will encourage stakeholders in environmental science to explore the connections of their work with others (rather than their specific domain, discipline, model, or method) and create innovative designs of DRI tools with these stakeholders that enable an interdisciplinary and systems approach to environmental challenges. This will help expose the relationships and feedbacks between stakeholders’ research and offer new insights into interventions. Moreover, by adopting a systems-thinking lens to DRI, we will also account for the impacts of the DRI itself by exploring DRI’s relationships with society and the environment. These include, for example, equality, diversity and inclusion considerations that may arise from our agile co-design insights, the financial and research culture implications for DRI’s support long-term, and DRI’s environmental sustainability, building on prior work in this domain (e.g., UKRI’s Net Zero DRI).

### Establishing a (federated) asset commons

Establishing an asset commons is critical to many of the principles above, which we define as: “A common place for supporting asset discovery, access, interoperability, and asset re-use (cf. the FAIR Principles), tailored for a community and managed by that community for the common good.” This extends data commons concepts to a broader asset commons, bringing FAIR to all aspects of environmental research; it also emphasizes the importance of a community-led approach whereby communities are responsible for determining how assets should be standardized and governed. Our view of implementing the commons is consistent with Grossman’s narrow middle architecture whereby the fewest possible core services are identified and applying standardization to these core services is prioritized.^10^ This architectural pattern offers a pragmatic and minimalist approach to standardization, focusing on this core and enabling innovation and diversity at the endpoints, including in the development of tools for data input, curation, analysis, and visualization.^10^ Using this as a commons implementation offers simplicity of standardization across assets, establishing a foundation for communities to integrate an ecosystem of assets^11^ and enabling a federated commons to be possible for cross-community collaborations and systemic science. It also addresses issues of alternative architectural models (e.g., individual thematic databases with related data portals or generalized data lakes), such as addressing the downstream burden of data integration across many data types inherent in data lakes.^12^ This vision aligns with other international initiatives, such as the Australian Research Data Commons.^2^

### Building effective teams for transdisciplinary DRI research

Innovating DRI for environmental science is a multifaceted effort that requires expertise in different domains and the establishment of a collaborative team for transdisciplinary DRI research and development. For example, to engage with stakeholders for DRI co-design and consider DRI’s broader impacts, there should be skills within human-computer interaction, systems thinking, design, and environmental sustainability. To develop DRI based on stakeholder requirements and commons concepts in an agile process, there should be skills in agile software development and operations (DevOps), systems architecture, and software engineering research. To enact FAIR asset integration within DRI, there should be expertise in semantic web concepts (e.g., vocabularies, ontologies), data science, asset stewardship, and impact tracking. Cross-cutting all role responsibilities is the need for team members to communicate well internally and externally about the DRI—liaising with communities (e.g., for co-design and training) to ensure the principles are realized—as well as managers that embrace complex transdisciplinary research and provide effective working cultures for team members to develop, share best practices, and learn from each other. This builds the foundations necessary for realizing the future of DRI for environmental science.

### Concluding remarks

There are numerous challenges to DRI for environmental science because of the changing nature of this science as well as innovations in digital technology. Yet, although challenging, the required future of DRI for environmental science pose exciting opportunities to advance data collection, management, analysis, insights, decisions, and actions for urgently addressing the climate and ecological crises. With our set of principles defining these DRI futures, we have shared our multi-dimensional approach to researching and developing DRI toward these futures. By sharing this research approach, we are excited to work with the community to change the status quo on digital technology in environmental science, explore the governance mechanisms that are ultimately required at its core to ensure the resultant DRI works for the community, and enable the DRI advancements required in this domain for the realization of an environmentally sustainable future.

## Acknowledgments

We thank the UKRI Natural Environment Research Council (NERC) for supporting numerous projects that underpin our DRI research and development at UKCEH: UK-SCAPE (NE/R016429/1), NERC-EDS (NE/Y001729/1), EDS Enhancement Project (EDS UKRI DRI Phase 1b), Economics of Biodiversity (NE/X002233/1), and pIMFe (NE/X016765/1). We thank the rest of the Environmental Data Science team at UKCEH as well as the wider DRI and environmental science communities that we have collaborated with in our DRI-related projects; we look forward to further collaboration for DRI for environmental science and to enhance the research approach and principles we have explored in this opinion paper.

## Author contributions

Conceptualization, K.W., F.S., G.S.B., S.R., and J.W.; investigation, K.W., F.S., G.S.B., S.R., and J.W.; writing – original draft, K.W., F.S., G.S.B., S.R., and J.W.; writing – review & editing, K.W., F.S., G.S.B., S.R., and J.W.; visualization, F.S.; supervision, G.S.B., S.R., and J.W.; project administration, K.W.; funding acquisition, G.S.B., S.R., and J.W.

## Declaration of interests

All authors currently work on enhancing digital research infrastructure for environmental science at the UK Centre for Ecology & Hydrology. Gordon S. Blair is also a distinguished professor of distributed systems at Lancaster University and a member of the *Patterns* advisory board.
